# A Study of the Mean Platelet Volume and Plasma Fibrinogen in Type Two Diabetes Mellitus Patients Versus Healthy Controls and Their Role as Early Markers of Diabetic Microvascular Complications

**DOI:** 10.7759/cureus.65458

**Published:** 2024-07-26

**Authors:** Sindhuri Goud Nimmala, Vijayashree S Gokhale, Ponvijaya Yadav, Sangram Mangudkar, Satbir Malik

**Affiliations:** 1 Department of General Medicine, Dr. D. Y. Patil Medical College, Hospital and Research Centre, Dr. D. Y. Patil Vidyapeeth, Pune, IND

**Keywords:** mean platelet volume (mpv), thrombogenecity, fibrinogen, platelet indices, diabetes mellitus type 2

## Abstract

Background

Mean platelet volume (MPV) is considered an emerging biological marker of platelet function and activity. Higher MPV has been scientifically linked to diabetes mellitus, metabolic syndrome, stroke, and coronary artery disease. Plasma fibrinogen is a circulating glycoprotein, serving as an acute inflammatory marker ultimately leading to enhanced atherogenic plaque formation. We conducted this study to evaluate the crucial role of MPV and plasma fibrinogen, which showed elevated levels in diabetes mellitus patients compared to non-diabetic healthy individuals. This study also elaborates on the pivotal role that MPV and plasma fibrinogen levels play in the pathogenesis of microvascular complications, which progress and eventually lead to mortality in patients with type 2 diabetes mellitus.

Methodology

This study is a single-center hospital-based study including 120 type 2 diabetes mellitus patients and 120 healthy non-diabetic individuals. It is a cross-sectional and observational study. The study was conducted over a period of one and a half years in a medical college and hospital in a semi-urban locality in Western Maharashtra, India. We obtained informed written consent from the patients. All patients underwent thorough clinical assessment, and data were collected using proformas, which were later tabulated and entered in Microsoft Excel sheets. Later, the statistical data analysis was performed. Plasma fibrinogen was performed by photo-optical clot detection. MPV was analyzed by coulter principle in the central laboratory department of the parent institute.

Patients above 18 years with cases of type 2 diabetes mellitus with or without any related complications, while the controls are healthy non-diabetic individuals attending the outpatient and inpatient departments of General Medicine. We excluded patients under the age of 18 years, those diagnosed with type 1 diabetes mellitus, hematological conditions associated with anemia and abnormal platelet counts, pregnant females, any acute or chronic infections, patients currently on antiplatelet medication and other drugs affecting the platelets, and all critical patients.

Results

The majority of patients in our study were in the age group of 41-50 years, with 49.2% having one or more microvascular complications of diabetes mellitus. In our study, out of 120 cases, 3.3% and 23.3% had raised MPV and fibrinogen levels, respectively, above the normal range. When compared with males and females, there was no statistically significant difference in the mean value of MPV and fibrinogen. On the t-test (p < 0.05), there was a statistically significant difference in the mean value of MPV and fibrinogen level between diabetics with and without microvascular complications. The t-test (p < 0.05) showed that there was a statistically significant difference among cases in the mean values of MPV and plasma fibrinogen in relation to retinopathy, nephropathy, and neuropathy, which are all microvascular complications of diabetes.

Conclusion

The study reveals higher levels of MPV and fibrinogen in diabetic patients compared to non-diabetic healthy individuals. In addition, higher levels of MPV and fibrinogen were present in patients with microvascular complications, correlated with age and diabetes duration.

## Introduction

Diabetes mellitus is a major health problem in India. Microvascular complications such as diabetic retinopathy, nephropathy, and neuropathy, along with macrovascular complications such as cardiovascular, cerebrovascular, and peripheral arterial disease, contribute significantly to the burden of type 2 diabetes mellitus. Ongoing evidence suggests that persistent subclinical inflammation serves as a trigger for the origin of type 2 diabetes mellitus. In our study, we selected the mean platelet volume (MPV) as a biological marker of platelet function and plasma fibrinogen as a predictor for inflammation of the endothelium and the development of atherosclerosis, which lead to the majority of diabetic microvascular complications [1,2].

Platelet indices, which are critical for maintaining normal homeostasis integrity [3], indicate platelet function. The MPV is considered an emerging biological marker of platelet function and activity. Studies have shown that platelet and inflammatory markers play a significant role in the increased susceptibility of diabetics to developing microvascular and macrovascular diseases [4,5].

Fibrinogen, a circulating glycoprotein in plasma in the form of a dimer, is composed of three pairs of polypeptide chains. It is an acute inflammatory marker that contributes to atheromatous plaque formation. There are numerous compelling studies that have established that fibrinogen plays a critical role in both the initial formation of plaque and the long-term effects of cardiovascular disease [6,7]. Monocytes that invade the plaque undergo a process of differentiation and transform into macrophages. These macrophages then release cytokines, specifically interleukin 6, which induce an abrupt spike in the plasma levels of fibrinogen. The current research intends to add data on the usefulness of MPV and fibrinogen as early predictors for detecting microvascular complications of diabetes mellitus.

Part of this study was presented as a research paper at the 13th Annual Conference of the Maharashtra Association of Physicians (MAPCON) on November 2-5, 2023, in Nagpur, Maharashtra, India.

## Materials and methods

Study design and setting

We conducted a cross-sectional and observational study on 120 individuals diagnosed with type 2 diabetes mellitus and 120 controls who are healthy, non-diabetic individuals. We performed it over a period of one and a half years at Dr. D. Y. Patil Medical College, Hospital and Research Centre, Pune. The hospital is located in a semi-urban locality in Western Maharashtra, India. Approval from the Institutional Ethics Sub-Committee of Dr. D. Y. Patil Medical College, Hospital and Research Centre, Pimpri, Pune, was obtained (approval IESC/PGS/2022/12). We obtained informed written consent from the patients. We collected data using proformas from outpatient and inpatient departments.

Inclusion criteria

Patients above 18 years with cases of type 2 diabetes mellitus with or without any related complications were included, while the controls are healthy non-diabetic individuals attending the outpatient and inpatient departments of General Medicine.

Exclusion criteria

We excluded patients under the age of 18 years, those diagnosed with type 1 diabetes mellitus, hematological conditions associated with anemia and abnormal platelet counts, pregnant females, any acute or chronic infections, patients currently on antiplatelet medication and other drugs affecting the platelets, and all critical patients who have been admitted to the intensive care unit.

Data and sample collection

As part of our study, we evaluated each patient with a comprehensive, detailed history, clinical examination, and basic laboratory investigations. Routine investigations done as a part of our study comprised of fasting and postprandial blood sugar, HbA1C, hemogram with MPV, plasma fibrinogen, electrocardiography, fasting lipid profile, renal function test, and urine albumin creatinine ratio for early nephropathy. We conducted fundus examinations to assess diabetic retinopathy and nerve conduction studies to evaluate diabetic neuropathy. Plasma fibrinogen was detected by photo-optical clot detection and MPV using the Coulter principle and microscopy in the central laboratory department of the parent institute. We collected and analyzed about 5 ml of venous blood using an ethylene di-amine-tetra-acetic acid (EDTA) vacutainer. Simultaneously, blood samples were obtained and examined for estimating fasting and postprandial blood glucose concentrations by the glucose-oxidase method and that of HbA1c by high-performance liquid chromatography.

Statistical analysis

Data entry and tracking were performed using Microsoft Excel (Microsft Corporation, United States). We analyzed the data entry using IBM SPSS Statistics for Windows, Version 26.0 (released 2019, IBM Corp., Armonk, NY). In all applied statistical tests, a p-value of <0.05 was considered a statistically significant value at a 95% confidence interval. We express categorical variables in terms of frequency and percentage and continuous variables in terms of mean and SD. We analyzed associations between different study parameters using the chi-square test or Fisher's exact test. To determine the significance of the mean pattern of parameters in relation to the diabetic and control groups, we applied a two-tailed Student's t-test. In all the applied statistical tests, a p-value of <0.05 was considered a statistically significant value at a 95% confidence interval.

## Results

Table 1 shows the age-wise distribution of the study participants. There were a total of 240 participants distributed in cases (type 2 diabetes mellitus patients) and controls (healthy non-diabetic individuals) in equal proportion (1:1). Among the cases, age-wise distribution found that the majority were in the age group of 41-50 years (27.5%), followed by 51-60 years (23.2%), 61-70 years (23.2%), below 40 years (16.7%), and lastly above 70 years (9.2%). The mean age of the study cases was 54.06 ± 12.4 years, and the age range was 30-84 years.

**Table 1:** Age-wise distribution of the study participants

Age group	Cases	Controls	Total
<40 years	20 (16.7%)	38 (31.7%)	58 (24.2%)
41-50 years	33 (27.5%)	32 (26.7%)	65 (27.1%)
51-60 years	28 (23.3%)	22 (18.3%)	50 (20.8%)
61-70 years	28 (23.3%)	20 (16.7%)	48 (20.0%)
>70 years	11 (9.2%)	8 (6.7%)	19 (7.9%)
Total	120	120	240

Table 2 shows the gender-wise distribution of the study participants. Among the cases, there was a male predominance (60% vs. 40%) male-to-female (M:F) ratio was 1.5:1. Among the controls, there was an almost equal distribution of males and females (50.8% vs. 49.2%).

**Table 2:** Gender-wise distribution among the cases and controls.

Gender	Cases	Controls	Total
Female	48 (40%)	59 (49.2%)	107 (44.6%)
Male	72 (60%)	61 (50.8%)	133 (55.4%)
Total	120	120	240

Table 3 shows the distribution of diabetes duration in the study participants. Out of 120 type 2 diabetes mellitus patients, 10% were new-onset cases; the majority (40.8%) had a duration of one to five years, followed by five to 10 years (26.7%), >10 years (12.5%), and less than a year (10%). The mean duration of diabetes mellitus was 5.04 ± 4.4 years and ranged from 0 to 20 years.

**Table 3:** Distribution of diabetes duration among the cases.

Diabetes mellitus duration	Frequency	Percent
New onset	12	10
Up to one year	12	10
One to five years	49	40.8
Five to 10 years	32	26.7
>10 years	15	12.5
Total	120	100

Table 4 shows that out of 120 cases, 59 (49.2%) had one or more microvascular complications of diabetes. Among the microvascular complications of diabetes mellitus, the most commonly observed was retinopathy (37.5%), followed by nephropathy (29.1%) and neuropathy (16.7%). Here, each study participant may have one or more microvascular complications simultaneously.

**Table 4:** Diabetes mellitus study group with and without microvascular complications.

Diabetes complications	Frequency	Percent
No complications	61	50.8
Microvascular complications	59	49.2
Retinopathy	45	37.5
Nephropathy	35	29.1
Neuropathy	20	16.7

Table 5 shows that out of 120 type 2 diabetes mellitus cases, 3.3% had raised MPV above the normal laboratory level. The mean MPV was 9.09 ± 1.34 femtoliter (fl) and had a statistical range of 6.3-13.1 fl.

**Table 5:** Mean platelet volume (MPV) levels among the cases

MPV (fl)	Frequency	Percentage
Raised (>11.7 fl)	4	3.3
Normal	116	96.7

Table 6 shows that out of 120 type 2 diabetes mellitus cases, 23.3% had raised fibrinogen above the normal laboratory level. The mean fibrinogen level was 334.14 ± 99.50 mg/dl, and the range was 159-501 mg/dl statistically.

**Table 6:** Fibrinogen levels among the cases

Fibrinogen (mg/dl)	Frequency	Percentage
Raised (>400)	28	23.3
Normal	92	76.6

Table 7 shows that there was a statistically significant difference in the mean values of various continuous study variables, i.e., MPV and plasma fibrinogen, on the analysis using the t-test (p-value < 0.05).

**Table 7:** Comparison of the mean values of the study variables among the cases and controls The mean values of the mean platelet volume (MPV) and plasma fibrinogen levels were analyzed using the T-test (p-value < 0.05).

Variables	Cases (mean ± SD)	Controls (mean ± SD)	P-value
MPV (fl)	9.09 ± 1.37	7.95 ± 0.98	0.001
Fibrinogen (mg/dl)	334.14 ± 99.50	209.31 ± 51.25	0.001

Table 8 includes 120 diabetic patients in the case study group, which showed that there was no statistically significant difference in the mean values of the MPV and plasma fibrinogen among the males and females when analyzed using the T-test (p-value > 0.05).

**Table 8:** Comparison of the mean values of the mean platelet volume (MPV) and fibrinogen among the males and females in the case group The mean values of the MPV and plasma fibrinogen were analyzed using the T-test (p-value < 0.05).

Variables	Males (mean ± SD)	Females (mean ± SD)	P-value
MPV (fl)	9.014 ± 1.45	9.21 ± 1.24	0.44
Fibrinogen (mg/dl)	332.28 ± 106.05	336.94 ± 89.79	0.803

As shown in Table 9, the mean levels of MPV and plasma fibrinogen in 120 cases with diabetes mellitus were statistically different between those with microvascular complications and those without microvascular complications when the T-test was used (p-value < 0.05).

**Table 9:** Comparison of the study variables' mean values in relation to diabetes mellitus microvascular complications. The mean values of the mean platelet volume (MPV) and plasma fibrinogen levels were analyzed using the T-test (p-value < 0.05).

variables	Microvascular complication (mean ± SD)	No microvascular complication (mean ± SD)	P-value
MPV (fl)	9.73 ± 1.30	8.48 ± 1.16	0.001
Fibrinogen (mg/dl)	374.17 ± 85.66	295.43 ± 97.18	0.001

Table 10 depicts that there was a statistically significant difference in the mean value of the MPV (fl) in relation to various diabetic microvascular complications, i.e., retinopathy, nephropathy, and neuropathy, on analysis using the T-test (p < 0.05) among the 120 cases.

**Table 10:** Comparison of the mean value of the mean platelet volume (MPV) in relation to diabetic microvascular complications The mean value of the MPV was analyzed using the T-test (p-value < 0.05).

Variables		MPV (mean ± SD) fl	P-value
Retinopathy	Present	7.01 ± 5.09	0.001
	Absent	3.86 ± 3.62	
Nephropathy	Present	9.84 ± 1.25	0.001
	Absent	8.77 ± 1.3	
Neuropathy	Present	10.08 ± 1.12	0.001
	Absent	8.89 ± 1.33	

Table 11 shows a statistically significant difference in the mean value of plasma fibrinogen (mg/dl) in relation to various diabetic microvascular complications, i.e., retinopathy, nephropathy, and neuropathy, based on analysis using the T-test (p < 0.05) among the 120 cases.

**Table 11:** Comparison of the mean value of plasma fibrinogen in relation to diabetic microvascular complications The mean value of the plasma fibrinogen levels was analyzed using the T-test (p-value < 0.05).

Variables		Fibrinogen (mean ± SD) mg/dl	P-value
Retinopathy	Present	380.27 ± 86.56	0.001
	Absent	308.89 ± 96.97	
Nephropathy	Present	408.41 ± 75.9	0.001
	Absent	306.04 ± 92.24	
Neuropathy	Present	372.35 ± 63.83	0.001
	Absent	326.5 ± 103.75	

## Discussion

Metabolic abnormalities in individuals with diabetes mellitus cause platelets to become larger and more active and to release a greater number of dense granules. This leads to an increased tendency for thrombotic events, which can result in both macrovascular and microvascular complications. These consequences contribute to higher rates of morbidity and mortality [8,9]. Endothelial dysfunction has been identified as an independent risk factor for cardiovascular morbidity in patients with type 2 diabetes mellitus, and fibrinogen is an early indicator of inflammation. There is evidence indicating that hyperfibrinogenemia and its consequences are caused by inadequate glycemic management. This study was aimed at finding a correlation between mean MPV and fibrinogen levels and glycemic control in type 2 diabetes mellitus cases admitted to our hospital, and we also tried to find differences in mean MPV and fibrinogen among type 2 diabetes mellitus cases and healthy controls.

We distributed a total of 240 participants in our study evenly between cases (type 2 diabetes mellitus patients) and controls (healthy patients) at a ratio of 1:1. The age-wise distribution found that the majority were in the age group of 41-50 years (27.5%), followed by 51-60 years (23.2%) and 61-70 years (23.2%). Out of 120 cases, 20 (16.7%) were below the age of 40, and 11 (9.2%) were >70 years old. The mean age of the study cases was 54.06 ± 12.4 years, and the age range was 30-84 years. There was male predominance in the cases group (60% vs. 40%), and the M: F ratio was 1.5:1. Among the controls, there was an almost equal distribution of males and females (50.8 vs. 49.2%). These results are in agreement with a study by Kim ES et al. [5] in Incheon St. Mary's Hospital (2015), where a cross-sectional study involved 1205 patients with type 2 diabetes mellitus with a mean age of 58.9 ± 10.7 years. In Bembde AS et al.'s study [10] in India (2012), a case-control study of 100 type 2 diabetes mellitus patients with age-matched controls had a mean age of diabetes of 56.4 years. Mahendra JV et al.'s study [11] (2015) in Karnataka, India, was a cross-sectional study of 100 type 2 diabetes mellitus patients.

In our study, out of 120 type 2 diabetes mellitus cases, 12 (10%) were new-onset cases; the majority (40.8%) had a duration of one to five years, followed by five to 10 years (26.7%), 12.5% had a duration >10 years, and 10% had a duration of less than a year. The mean duration of diabetes was 5.04 ± 4.4 years and ranged from 0 to 20 years.

In our study, out of 120 type 2 diabetes mellitus cases, 3.3% had raised MPV above the normal range. The mean MPV was 9.09 ± 1.34, with a range of 6.3-13.1. There was a statistically significant difference in the mean value of MPV among the cases and controls (9.09 ± 1.37 vs. 7.95 ± 0.98). In a study conducted by Agarwal P et al. [12] (2023) in India and another one by Bhattacharjee P et al.(2023) [13], a case-control study on 100 diabetic patients who were age- and sex-matched with 100 nondiabetic controls in North India was performed; both results were similar and found that platelet indices, i.e., P-LCR (platelet large cell ratio), MPV, and PDW (platelet distribution width), were notably higher in the diabetic patients when contrasted with individuals without diabetes.

Poor glycemic control in patients with diabetes mellitus and persistently high blood sugar levels induces a prothrombotic state. The process of glycation of platelet surface proteins enhances adhesion between platelets. Platelet activation is triggered by both acute and chronic conditions of hyperglycemia. Previous literature has proven that the MPV is elevated in individuals with diabetes compared to those without diabetes [14,15]. Large, activated platelets adhere to injured endothelial cells and accumulate due to elevated levels of advanced glycation end products, increased production of von Willebrand factors by the damaged endothelial cells, and reduced sensitivity of platelets to nitric oxide in the bloodstream. As a consequence, thrombus formation and embolization of microcapillaries take place, leading to the emergence of vascular abnormalities. The statement suggests an association between the functioning of platelets and vascular injury in individuals with diabetes, which is the primary root cause of morbidity and mortality in this condition [16].

As a secondary objective, we sought to examine the correlation between MPV mean values and diabetes complications. The mean value difference for MPV was observed in relation to diabetic complications versus the non-complication group (9.73 ± 1.30 vs. 8.48 ±1.16), which correlates with the findings of Papanas et al.'s 2004 study, Dindar et al.'s 2013 study, and Ates et al.'s 2009 study [17,18,19]. It is anticipated that persistently elevated blood sugar levels and an increased volume of platelets play a crucial role in the development of diabetes-related complications, as this finding clearly highlights the issue. Multiple risk factors regulate the pathogenesis of microvascular complications in persistently elevated hyperglycemic individuals. The fundamental processes involved in diabetes complications include the synthesis of advanced products of glycation, the stimulation of protein kinase C, and disruptions in polyol mechanisms. The occurrence of larger platelets, which are younger, more reactive, and aggregable, possess denser granules, and release higher levels of thromboxane A2, serotonin, and β-thromboglobulin than smaller platelets, is believed to indicate an increase in MPV among individuals who suffer from diabetes mellitus [20,21,22]. Each of these factors can induce a pro-coagulant response and lead to thrombotic vascular consequences. It indicates a correlation between the MPV and microvascular diabetic complications, such as different stages of retinopathy. Therefore, in patients with diabetes mellitus, platelets with increased MPV could potentially have a crucial part in signaling the onset of retinopathy and other vascular problems.

In our study, the mean fibrinogen level in all diabetes mellitus patients turned out to be elevated, 334.14 ± 99.50 mg/dl in cases, in contrast to 209.31 ± 51.25 mg/dl in the control group, with a statistically significant difference (p = 0.0001). Moreover, on comparing the mean fibrinogen level of type 2 diabetes mellitus patients with complications (374.17 ± 85.66) versus those without complications (295.43 ± 97.18), we observed that the difference was statistically significant (p = 0.0001). Our study's findings, which showed higher levels of fibrinogen in individuals with type 2 diabetes mellitus, coincide with those reported by Bembde AS et al. [10] (2012) in India, Mahendra JV et al. [11] (2015) in Karnataka, India, and Ghongade PV et al. (2020) in India [23]. In the above-mentioned studies, the mean fibrinogen value in complicated cases was found to be significantly greater than the mean fibrinogen value in uncomplicated type 2 diabetes. Thus, we came to the conclusion that the severity of type 2 diabetes mellitus is linked to the levels of plasma fibrinogen level.

In our study, out of 120 cases, 59 (49.2%) had one or more microvascular complications of diabetes. The most common observed was retinopathy (37.5%), followed by nephropathy (29.1%) and neuropathy (16.7%). In our study, there was an association between high fibrinogen levels and various microvascular complications. The findings of our study are similar to all those of Bembde AS et al. [10] (2012) in India, which is a case-control study of 100 type 2 diabetes mellitus with age-matched controls; Mahendra JV et al. [11] (2015) in Karnataka, India, which is a cross-sectional study of 100 type 2 diabetes mellitus; Razak MK and Sultan AA et al. [24] (2019) in Iraq, which is a study involving 50 type 2 diabetes mellitus and 30 controls; and Patel HV et al. [25] (2023), which is a cross-sectional study of 114 type 2 diabetes mellitus cases.

In agreement with the present study, Khan et al.'s [26] (2005) study in Pakistan, consisting of 120 subjects of diabetes patients with complications, found that plasma viscosity and fibrinogen levels were both markedly higher compared to those without complications and healthy control subjects. It is also important to note that fibrinogen is an important predictor of cardiovascular disease in the general population, and those with type 2 diabetes mellitus have been reported to have higher fibrinogen levels [27].

Fibrinogen serves an essential role in the coagulation cascade along with being a major determinant of blood flow and blood viscosity. Multiple variables, including age, gender, obesity, and cigarette smoking, can affect fibrinogen levels. Conditions such as diabetes, inflammatory disorders, and cardiovascular disease also exhibit higher levels of fibrinogen. Elevated levels of fibrinogen have been implicated in an increased susceptibility to a spectrum of health disorders, especially cardiovascular disease, deep vein thrombosis, pulmonary embolism, and stroke.

Low-grade inflammation and elevated cytokines, particularly interleukin 6, may accompany hyperfibrinogenemia in diabetes mellitus. These cytokines stimulate hepatocytes to generate fibrinogen, indicating a key connection between hypercoagulability and the systemic inflammatory response. In addition, elevated blood sugar levels along with insulin resistance in the peripheral tissues exert a significant influence on fibrinogen levels. The levels of fibrinogen are related to insulin levels in individuals who are non-diabetic as well. Individuals with diabetes experience high levels of fibrinogen due to a procoagulant state. This occurs because there are greater levels of coagulation proteins in the cascade, such as fibrinogen, von Willebrand factor, factor VII, plasminogen activator inhibitor 1, and antithrombin products. Researchers have linked all these variables to both microvascular and macrovascular complications, as well as glycemic control [28,29,30]. According to reports, diabetes mellitus patients experience hyper-fibrinogenemia due to an increase in fibrinogen synthesis without a corresponding increase in its clearance. This abnormality is associated with insulin deficiency and has been corrected with insulin, suggesting that hyperfibrinogenemia is an expression of poor glycemic control. Hence, the idea that inadequate glycemic control might result in thrombophilia, a disorder that may contribute to elevated cardiovascular risk in patients with diabetes mellitus, is supported by both epidemiologic and clinical data. Our findings also showed that elevated levels of fibrinogen have been linked to an increased risk of developing diabetes and its complications.

Limitations of the study

The study was conducted at a single center; hence, the sample size was too small, and the diversity of the sample was limited. A hypothesis cannot be made for the generalization of the study findings. Further multicentric studies with a large sample size are required in the future for the implication of study findings on a larger scale.

## Conclusions

Based on our findings, type 2 diabetes mellitus patients have higher MPV and higher plasma fibrinogen levels when compared to healthy, non-diabetic controls. Our results also imply that patients with diabetic microvascular complications have higher levels of MPV and higher plasma fibrinogen levels. This aids in early detection, which is critical for reducing morbidity and healthcare costs. By analyzing MPV and fibrinogen levels in type 2 diabetic patients, we may counsel them to lower their higher glycemic levels to a controlled diabetes mellitus category (HbA1c<7) to prevent devastating complications.
